# The SciQA Scientific Question Answering Benchmark for Scholarly Knowledge

**DOI:** 10.1038/s41598-023-33607-z

**Published:** 2023-05-04

**Authors:** Sören Auer, Dante A. C. Barone, Cassiano Bartz, Eduardo G. Cortes, Mohamad Yaser Jaradeh, Oliver Karras, Manolis Koubarakis, Dmitry Mouromtsev, Dmitrii Pliukhin, Daniil Radyush, Ivan Shilin, Markus Stocker, Eleni Tsalapati

**Affiliations:** 1 TIB—Leibniz Information Centre for Science and Technology, Hannover, Germany; 2 L3S Research Center, Leibniz University Hannover, Hannover, Germany; 3 Institute of Informatics, Federal University of Rio Grande do Sul, Porto Alegre, Brazil; 4 Department of Informatics and Telecommunications, National and Kapodistrian University of Athens, Athens, Greece; 5 Laboratory of Information Science and Semantic Technologies, ITMO University, St. Petersburg, Russia

**Keywords:** Computer science, Information technology, Scientific data

## Abstract

Knowledge graphs have gained increasing popularity in the last decade in science and technology. However, knowledge graphs are currently relatively simple to moderate semantic structures that are mainly a collection of factual statements. Question answering (QA) benchmarks and systems were so far mainly geared towards encyclopedic knowledge graphs such as DBpedia and Wikidata. We present SciQA a scientific QA benchmark for scholarly knowledge. The benchmark leverages the Open Research Knowledge Graph (ORKG) which includes almost 170,000 resources describing research contributions of almost 15,000 scholarly articles from 709 research fields. Following a bottom-up methodology, we first manually developed a set of 100 complex questions that can be answered using this knowledge graph. Furthermore, we devised eight question templates with which we automatically generated further 2465 questions, that can also be answered with the ORKG. The questions cover a range of research fields and question types and are translated into corresponding SPARQL queries over the ORKG. Based on two preliminary evaluations, we show that the resulting SciQA benchmark represents a challenging task for next-generation QA systems. This task is part of the open competitions at the 22nd International Semantic Web Conference 2023 as the Scholarly Question Answering over Linked Data (QALD) Challenge.

## Introduction

Knowledge graphs have gained increasing popularity in the last decade in science and technology. They enable a versatile and evolving semantic representation of knowledge at the crossroads of various

- levels of information structuring: unstructured, semi-structured, structured;
- levels of abstraction: conceptual vs. operational;
- knowledge representation formalisms: graphs, facts, entity-relationship, logic; and
- technology ecosystems.

However, most publicly available knowledge graphs, such as DBpedia or Wikidata, are relatively simple to moderate semantic structures^1^. Although they vary in content, size, coverage, and overlap, they all primarily represent a collection of factual statements arranged in entity descriptions, possibly enriched by class hierarchies and corresponding property definitions. Question answering (QA) benchmarks and systems were so far geared primarily towards encyclopedic knowledge graphs such as DBpedia and Wikidata^2,3^. Currently, a new type of knowledge graph, called research knowledge graphs, is emerging whose contents are bibliographic metadata and scientific elements, such as ideas, theories, approaches, and claims as they are conveyed in scholarly contributions^4,5^ or OMICS data structures for personalized medicine^6^. These novel research knowledge graphs increasingly intertwine three previously largely isolated aspects: semantic representations (semantic intelligence), machine learning (machine intelligence), and crowd and expert sourcing (human intelligence). In particular, scholarly communication is a more challenging application domain for QA due to:

1. The heterogeneity of knowledge representation;
2. The concept drift and knowledge evolution along with the scientific discourse;
3. The different knowledge granularity used to describe research contributions;
4. The novel knowledge structures that go beyond simple entity descriptions.

**Figure 1:**
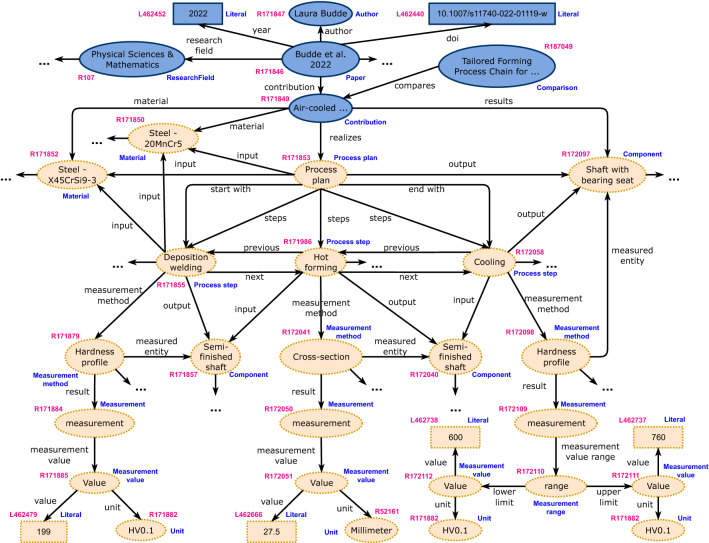
Concrete example of a paper^8^ described in the ORKG^9^: Blue shapes are resources and predicates of the ORKG schema. Yellow shapes are user-generated resources and predicates. The human-readable labels of the classes are represented in blue color. The IDs of the resources within the ORKG are shown in magenta.

We present SciQA a Scientific QA benchmark for scholarly knowledge. The benchmark leverages the Open Research Knowledge Graph (ORKG)^4,7^ (https://orkg.org) currently comprising almost 170,000 resources describing research contributions of almost 15,000 scholarly articles from 709 research fields. These research contributions contain, among other things, details about the research process, methods and materials used, and specific results. Figure 1 shows a concrete example of a paper by Budde et al.^8^ described in the ORKG^9^. This paper reports on four mechanical processes for manufacturing hybrid solid components. In Fig. 1, we show only parts of the description of one of the four processes described in the ORKG. Overall, each of the four descriptions includes details on the entire mechanical process concerning the individual steps, their sequence and, per step, the incoming and outgoing components, measurement methods, and measurement results.

Following a bottom-up methodology, we first manually developed a set of 100 questions that can be answered with the ORKG. Subsequently, we devised eight question templates with which we automatically generated further 2465 questions that also can be answered with ORKG. The handcrafted and autogenerated SciQA questions cover several research fields ranging from Computer Science, Engineering, Chemistry, and Geology in science and technology, over Immunology and Genetics in the life sciences to Economics and Urban Studies in the social sciences. The questions cover numerous question types, including non-factoid and factoid questions, and have corresponding SPARQL queries of different query shapes and triple pattern sizes. We translated the questions into SPARQL queries over the ORKG and provide a comprehensive set of related ground-truth query results.

We demonstrate the applicability and feasibility of the SciQA benchmark by presenting two preliminary evaluations, both targeting the 100 handcrafted questions. First, we present a proof-of-concept implementation of a scholarly QA system based on the JarvisQA system^10^. JarvisQA operates exclusively with tables or tabular views of knowledge graphs, and the autogenerated part of SciQA is not based on tables or tabular views. For this reason, the evaluation is performed only for the handcrafted part of SciQA. However, the JarvisQA system is only able to retrieve correct answers for a subset of handcrafted questions due to the more diverse data and question types in SciQA compared to the data JarvisQA was built on^10^. Second, we present initial insights into using the large language model (LLM) ChatGPT^11^ for answering the handcrafted questions. This evaluation aims to understand how well one of the current most famous LLMs is able to answer complex queries on scholarly knowledge (with superlatives, comparisons, etc.). In this evaluation, we also focused on the 100 hand-crafted questions to compare the results of JarvisQA with those of ChatGPT. In both preliminary evaluations, we found that the systems perform rather low. In the best-performing configuration ($Jarvis_{XLS2}$), the proof-of-concept implementation of JarvisQA was able to answer 52 questions with 12 correct answers. ChatGPT provided answers for 63 questions, of which only 14 answers were correct. These low numbers substantiate that answering questions about scholarly knowledge is a challenge for current QA systems and LLMs^12^. For this reason, we conclude that the SciQA benchmark represents a challenging task for next-generation QA systems, as QA systems must now also deal with scientific knowledge in addition to encyclopedic knowledge.

## Related work

The problem of answering questions expressed in natural language has received a lot of attention recently. Depending on the kind of system queries, e.g., text documents, knowledge graphs, relational databases, or image archives, benchmarks have been developed for evaluating the respective QA systems. Since the focus of this paper is on QA over scholarly knowledge graphs, we concentrate on QA benchmarks over knowledge graphs and linked data. An overview of relevant benchmarks is presented in Table 1.

**Table 1:** QA benchmark comparison (full comparison is available in the ORKG^13^).

QA Benchmarks	SciQA	LC-QuAD 2.0	QALD-9	Web-Questions-SP	Simple-Questions-Wikidata	Complex-Web-Questions
Questions	2565	30,000	408	4737	21,957	34,689
Domain	Scholarly communication	Encyclopedic				
Languages	English	English	Multi- language	English	English	English
Question types	Complex, factoid, non-factoid	Complex, factoid, non-factoid	Complex, non-factoid	Simple, factoid	Simple	Complex, factoid
Knowledge base	ORKG	Wikidata DBpedia	DBpedia	Freebase	Wikidata	Freebase
Formal language	SPARQL	SPARQL	SPARQL	SPARQL	SPARQL	SPARQL
Answers	$\checkmark$	$\times$	$\checkmark$	$\checkmark$	$\checkmark$	$\checkmark$
Paraphrases	$\checkmark$	$\checkmark$	$\times$	$\times$	$\times$	$\times$

One of the first datasets is WebQuestions^14^, which contains 5810 factoid question-answer pairs and is targeted at Freebase. It was created using the Google Suggest API to obtain questions that begin with a “wh”-word. 100K randomly selected questions were submitted to Amazon Mechanical Turk, asking workers to annotate the ones that can be answered by Freebase. In terms of structural complexity, WebQuestions is simple, as many questions only contain one class, one property, and one instance. In 2016, WebQuestions was extended to WebQuestionsSP^15^, by providing SPARQL queries for the 4737 questions that the annotators could fully process to find the answers.

The dataset SimpleQuestions^16^ also targets Freebase. It was created manually by English-speaking annotators and consists only of factoid questions. It is much larger than WebQuestions, containing 108,442 simple questions paired with their corresponding answers and explanations. Diefenbach et al.^17^ created the benchmark SimpleQuestionsWikidata by converting SimpleQuestions to target Wikidata.

The LC-QuAD dataset^18^ differs from the previous ones, as it includes not only simple, factoid questions but also complex ones, i.e., the respective SPARQL queries contain multiple triple patterns. The dataset contains 5000 pairs of questions-SPARQL queries targeting DBpedia. The questions were generated semi-automatically by extracting sub-graphs containing triples within a 2-hop distance from a seed entity. The generation of the SPARQL queries and questions was facilitated automatically, using templates, and, then, refined manually. After the development of LC-QuAD, its developers proceeded with the development of LC-QuAD 2.0^19^, which contains 30,000 questions, their paraphrases, and their corresponding SPARQL queries. LC-QuAD 2.0 targets both Wikidata and DBpedia 2018, and it was created similarly to LC-QuAD. LC-QuAD 2.0 also contains questions of higher complexity: Non-factoid questions, questions with qualifiers, aggregates, temporal aspects (as qualifiers), and superlatives. The benchmark ComplexWebQuestions^20^ (34,689 questions) has similar complexity: it contains composition questions, superlatives and comparatives. It was generated from WebQuestionsSP by sampling question-query pairs and automatically creating more complex SPARQL queries. From these queries, a set of questions was generated automatically by using 687 templates, and, then, reformulated by Amazon Mechanical Turk workers.

The benchmarks generated for the QALD challenges (http://qald.aksw.org/) are also of high complexity. The QALD- 10 benchmark, generated for testing in the latest challenge (NLIWoD, ESWC2022), contains 394 manually created Wikidata-based questions of varied complexity and each is annotated with a manually specified SPARQL query and its output. Each question may contain counts, superlatives, comparatives, and temporal aggregators. The questions are available in 4 different languages, i.a., English, German, Chinese, and Russian. The questions in English were collected from speakers with at least a C1-level language proficiency in accordance with the Common European Framework of Reference for Languages and, according to the participants, express real-world information needs. Native speakers translated the questions into other languages.

All benchmarks in this section target either Freebase, DBpedia, or Wikidata and thus mainly encyclopedic knowledge graphs. SciQA is the only benchmark with a focus essentially on scholarly knowledge. A major advantage of using ORKG as a basis is that it allows the generation of sophisticated queries, e.g., with superlatives and comparisons, over scholarly knowledge, as the ones presented in the SciQA Benchmark, and if necessary, provides the relevant evidence. This advantage is further enhanced by the class of “comparisons” among research outputs that the ORKG contains. Comparisons provide condensed overviews of the state-of-the-art for a particular research question. In this way, SciQA includes sophisticated questions over these comparisons, i.e., over aggregations of semantic contribution descriptions from various scientific papers. As our applicability and feasibility evaluation has shown, sophisticated QA systems and LLMs, like ChatGPT, have difficulties answering these types of questions that require scholarly knowledge. In fact, only 12 and 14 of the 100 handcrafted SciQA questions were answered correctly by the QA system and the large language model, respectively.

Regarding its structure, SciQA meets the current standard of QA benchmarks as it contains natural language questions, SPARQL queries, and answers. Finally, it is also the only benchmark that includes the features of the SPARQL queries, e.g., query shape, query components, and triple pattern sizes.

## The ORKG dataset

**Figure 2:**
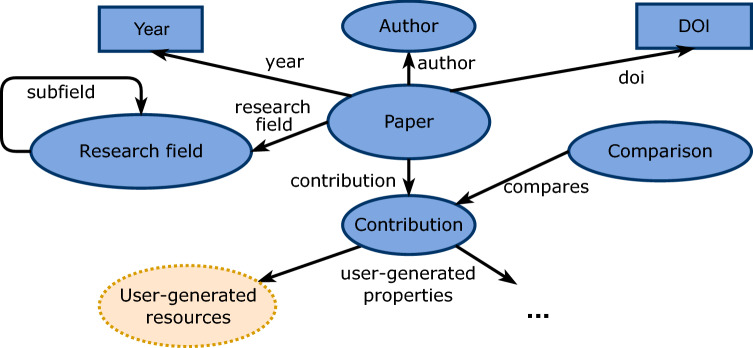
Basic ORKG schema for papers: Blue shapes are resources and predicates of the ORKG schema. Yellow shapes are user-generated resources and predicates. IDs and classes of resources and predicates are omitted for brevity.

The ORKG^4^ is a research knowledge graph that includes semantic descriptions of research articles and accompanying services (https://orkg.org) for the production, curation, and (re)use of this data. The structured knowledge in the ORKG is contributed, i.e., crowdsourced, by researchers and partially also automatically extracted from the literature or integrated from other resources comprising structured research contribution descriptions^21^. Figure 2 provides an overview of the core structure of ORKG. Each paper added to the ORKG contains its bibliographic metadata, i.e., authors, title, year of publication, DOI, research field, and the user-generated semantic description of its scientific contribution. In Fig. 1, we show a concrete example of a paper by Budde et al.^8^ described in the ORKG^9^.

The core entity in the ORKG is *contributions* presented in the form of research *papers*. A contribution is typically linked to a research field and problem, and its description includes several properties that are specific to the research field or problem. There is no predefined, fixed set of properties to describe research contributions, but these properties can be defined on the fly by the curators. On the one hand, this openness and extensibility allow selecting and defining a very appropriate knowledge representation for the research paper at hand. On the other hand, it brings significant challenges for potential QA applications. Based on described papers and their contributions, contributions dealing with a specific research problem in scholarly literature can be compared in so-called *comparisons*. Comparisons are tabular representations of the properties of all compared contributions^22^. Such comparisons provide an overview of key information about a research problem across dozens or hundreds of contributions. In this way, comparisons are a valuable tool, for example, to determine the leading sorting algorithm or to find out how dangerous a virus is compared to other viruses.

Table 2 provides some statistics about the ORKG knowledge graph. The ORKG is still relatively small compared to other knowledge graphs, which often include millions of entities^1^. However, we anticipate that these novel structures of scholarly knowledge in the ORKG already pose a challenging task for QA systems.

**Table 2:** Overview of the size of the ORKG knowledge graph based on the exported RDF dump^23^.

General values		Technical values	
Type	Number	Type	Number
Papers	14,538	Triples	1,133,217
Contributions	21,243	Classes	1621
Research problems	5065	Properties	6888
Research fields	709	Resources	169,967
Comparisons	1132	Literals	575,525

## Approach

In this section, we describe our approach to creating the SciQA benchmark. Creating a QA benchmark requires a collection of questions covering different aspects and scenarios on which a knowledge graph contains data. In particular, our approach consists of two main steps: (1) Manual creation of 100 handcrafted questions and (2) Automated generation of 2465 autogenerated questions. This approach is inspired by the procedures of related works that also combined the use of manual question creation and automated question generation^16,18–20^. To create the SciQA benchmark, we followed a detailed methodology that tackles the following key aspects: *Objectives*, *Data Structure*, and *Data Collection*.

**Figure 3:**
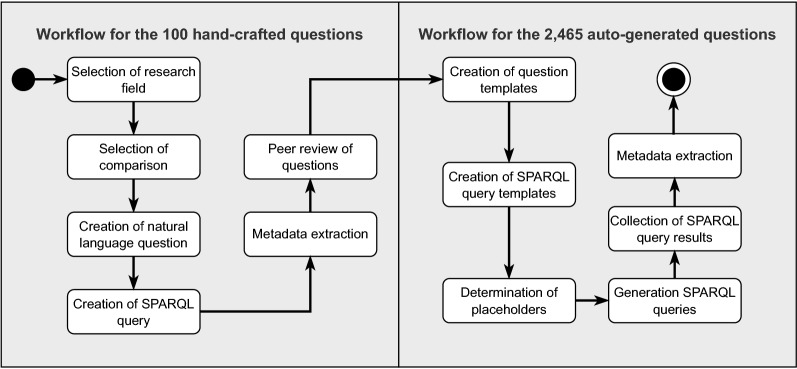
Detailed workflow for the development and generation of the SciQA benchmark.

1. *Objectives* The goal of the benchmark is not only to create a dataset but also to provide a variety of knowledge base scenarios, i.e., what questions can be asked on the knowledge graph or what type of use cases can utilize the data.
2. *Data Structure* The ORKG^4^ represents information as papers and a collection of contributions. Figure 2 depicts the core schema of the scholarly data in the ORKG that was followed to query data from the source.
3. *Data Collection* We performed the collection of the data in two main steps: (1) Manual creation and (2) Automated generation. Figure 3 illustrates the entire approach in the form of an activity diagram to highlight the main activities we undertook to create the SciQA benchmark.
  1. *Workflow for the 100 handcrafted questions*
    We started with the first workflow by selecting research fields and corresponding lists of ORKG comparisons^24^ in these fields to limit the scope of the data being queried. Subsequently, we defined several natural language questions according to different types, e.g., single comparison questions, True/False questions, aggregation questions (min, max, average), etc. For each natural language question, we created one SPARQL query in two variations (human-readable and machine-readable). As the questions and queries were created, we collected associated metadata for them, e.g., type, query shape, etc. Finally, the natural language questions, the structured SPARQL queries, and the collected metadata were peer-reviewed multiple times for syntactic and semantic correctness.
    All 13 authors from five countries and three continents participated in this workflow to accommodate different perspectives. In addition, these researchers consulted domain experts from their personal networks, when available, to ensure that the questions created were relevant and important to the respective research fields and researchers. Furthermore, we leveraged the expertise of the 21 domain experts participating in the ORKG curation grants^25,26^ to create the relevant, realistic and useful questions.
  2. *Workflow for the 2465 autogenerated questions*
    We performed this second workflow to enrich the SciQA benchmark as, even though the 100 handcrafted questions were purposefully created, this number of questions is rather small for a benchmark. For this purpose, we expanded the SciQA benchmark through the integration with a set of automatically generated questions, which have been created using a structured approach that involves a combination of handcrafted questions and queries, and the utilization of a LLM (in this case GPT-3^27^). The objective of the autogenerated questions is to target specific parts of the ORKG by creating queries with placeholders that can be populated with various entities, thereby facilitating the generation of numerous natural language questions.
    To create the autogenerated questions, we followed a structured process. When creating the handcrafted questions, we observed that the data in the ORKG is very heterogeneous, which complicates the automatic generation of questions and queries. For this reason, we decided to set certain restrictions on the generation of the questions and queries. First, we decided to focus on a specific dataset from papers-with-code^28^ that is available in ORKG. Although this dataset belongs to only one research field (Computer Science), it is extensive, with 2236 papers (around 15% of the total number of papers in the ORKG) that are homogeneously described. This homogeneity is important as it facilitates the automatic generation of questions and queries. Second, we decided to focus on the questions and queries with the shape of a tree, the class which-what, and the type factoid to further narrow down the scope of the automatic generation. This shape, class, and type are the most common in the handcrafted questions and also match the nature of the selected papers-with-code data.
    Initially, we crafted a set of eight queries and 32 questions. For each query, we created one question manually and three variations using GPT-3^27^ with careful manual validation. Next, we collected all possible entities for the placeholders in the queries from the ORKG. We then filled the queries’ placeholders with all possible entities, selecting one question randomly for each query. Finally, we collected the results of the created queries and extracted metadata for the final set of questions.
    The addition of the autogenerated questions expands the SciQA dataset to a total of 2565 questions and queries, providing a larger corpus for training machine-learning-based question-answering systems. This approach can be particularly useful compared to relying solely on handcrafted questions, which are often limited in number and may not capture the full scope of the underlying data. By contrast, the use of machine-generated questions provides a more diverse and extensive set of questions that can help improve the accuracy and robustness of machine-learning models in answering questions on large knowledge graphs.

## SciQA benchmark

In this section, we provide an overview of the SciQA questions and their corresponding SPARQL queries. We first explain how we classified the questions to extract the metadata, before presenting some examples of handcrafted and autogenerated questions in more detail.

### Question classifications

An appropriate question typology helps to satisfy two main goals of QA benchmark development. Namely, (1) an extensive coverage of the different topics in various subject areas that appear in the knowledge graph, and (2) the validation of the patterns used for writing questions and queries to ensure a better and more balanced distribution of questions across the possible different types of information requested.

There are many existing approaches to defining taxonomies of question types. Wendy Lehnert^29^ proposed a conceptual taxonomy with 13 conceptual classes, e.g., causal antecedent, goal orientation, enablement, etc. Li and Roth^30^ developed a two-layered taxonomy based on the answer type semantics: six coarse classes (abbreviation, entity, description, human, location, numeric value) and 50 fine classes (subclasses of different coarse classes do not overlap). Singhal et al.^31^ designed a small set of simple answer types corresponding to question classes, words, and expected answer types: Person, Location, Organization, Date, Quantity, Duration, Linear Measure. For example, if a question starts with who or whom, its type will be Person. The system Quarc^32^ defines a question categorization based on the use of certain interrogative pronouns, e.g., who, what, when, where, or why. A similar approach was used by the system AskBill^33^, where eleven question types were defined with question patterns such as type “QTemporalAge” identified with pattern “How old/At (Which/What) age”.

Research data and their descriptions have a very complex structure and semantics. When developing questions to search for information within this data, it is useful to define the types of expected answers and the focus of the questions. The definition of necessary types of expected answers is based on the results of evaluation campaigns of QALD^34^ and analysis of characteristic problems associated with the task of mapping natural language to formal queries presented in Cimiano and Minock^35^. These problems include:

- Lexical ambiguities arise when one word can be interpreted in different ways, i.e., it can refer to different entities or concepts.
- Light expressions such as the verbs “to be” and “to have”, and the prepositions “of” and “with” either refer to an ontological property in a highly underspecified way or do not correspond to any property at all.
- Lexical gap between the user’s vocabulary and that of the ontology.
- Complex questions that can only be expressed using queries involving aggregation functions, comparisons, superlatives, and temporal reasoning.

The definition of the focus of a question makes the search for an answer more specific. Moldovan et al.^36^ defined the *question focus* as a word or sequence of words that indicate what information is being asked about in the question. Ferret et al.^37^ defined the question focus as “a noun phrase that is likely to be present in the answer” consisting of a head noun and a list of its modifiers. For example, the question “*What types of nanocarriers do have therapeutic effect*?” has the focus on “types of nanocarriers”. According to Mikhailian et al.^38^ there are two types of question foci:

1. Asking Point (AP), which is denoted explicitly, e.g., words “research problems” in the question “*What are the research problems Vernier Effect is related to*?”.
2. ExpectedAnswerType (EAT), is an implicit answer that can be inferred from the information provided by the question, e.g., answer type “person” is the EAT for the question “*Who are the authors of the SOSA ontology*?”.

For our methodology, we modified the approach of Moldovan et al.^36^ by combining the question types, e.g., WHAT, WHO, WHICH, etc., corresponding to classes from the ORKG schema, e.g., Paper, Problem, etc., and the question patterns that define the expected answer (BOOLEAN, WHAT-WHO, WHAT-WHEN, WHICH-WHERE, WHICH-WHAT, and WHO-WHAT). For instance, the question “*Who is the author of the most recent paper about insects*?” has the pattern WHO-WHAT. We also classified the questions according to the following dimensions:

- **ORKG-content** This classification is based on the structure of the ORKG schema.
  - Paper-based: Questions on the content of a single or multiple research papers, e.g., “*Which papers use DBLP as a dataset*?”.
  - Comparison-based: Questions on the content of a comparison, i.e., on the properties that the contributions participating in a comparison share, e.g., “*What is the most common knowledge representation method in Semantic Representations of Scholarly Communication*?”.
- **Question content** Following the approach of Mikhailian et al.^38^, we classify the questions into either factoid, i.e., AP, or non-factoid, i.e., EAT. Factoid questions assume an explicit AP mapping to the entities of the ORKG ontology. If the answer to a question requires inference of a sequence of facts, counting, or filtering, we consider such questions non-factoid. We further classify them according to superlatives, e.g., “*What is the most common lead compound in Anuran Antimicrobial Peptides Activity and Mechanism Against Different Biological Membranes*?”, negation questions, e.g., “*What percentage of comparisons lacks a class link?*”, questions with counts, e.g., “*What is the total number of species examined in Invasion Biology-Enemy release hypothesis*?”, ranking questions, i.e., asking for a min/max value, e.g., “*What is the maximum female percentage in Brief Psychotherapy for Depression Studies*?”, temporal questions, e.g., “*How many studies are published after 2019*?”, or a combination of various types of content, e.g., “*Which was the most popular approach for summarization until 2002*?”.

Finally, we characterized the questions based on important properties of their respective SPARQL queries:

- **Number of triple patterns** In contrast to simple questions, the SPARQL query of complex questions consists of more than a single triple pattern^18^. As presented in Tables 3 and 4, the dataset contains both simple and complex questions with up to 14 triple patterns.

**Table 3:** Overview of the SciQA handcrafted queries.

Characteristic	Number					
Research fields	48					
SPARQL queries	100					
Query shapes	Tree	Chain	Star	Forest	Edge	Cycle
	47	39	7	5	1	1
Query classes	Which–What	Boolean	What–When	What–Who	Who–What	Which–Where
	84	5	4	3	3	1
Query types	Factoid	Non-factoid	Superlative	Temporal	Count	
	61	39	26	3	7	
Query components	Min: 2, Med: 5, Max: 14					
Triple patterns	Min: 1, Med: 4, Max: 14

**Table 4:** Overview of the SciQA autogenerated queries.

Characteristic	Number					
Research fields	1					
SPARQL queries	2465					
Query shapes	Tree	Chain	Star	Forest	Edge	Cycle
	2465	0	0	0	0	0
Query classes	Which–What	Boolean	What–When	What–Who	Who–What	Which–Where
	2465	0	0	0	0	0
Query types	Factoid	Non-factoid	Superlative	Temporal	Count	
	2122	343	0	0	0	
Query components	Min: 4, Med: 6, Max: 13					
Triple patterns	Min: 6, Med: 6, Max: 11
- **Query shape**: We identified the shape (single edge, chain, star, cycle, tree, etc.) of the queries according to Bonifati et al.^39^. Note that the classification based on the number of triple patterns is incorporated in this classification, as simple questions can be classified as single-edge queries.
- **Query components** We have noted the keywords and operators that are used to build each query, for instance, SELECT, ASK, DESCRIBE, COUNT, REGEX, STR, FILTER. These components give an insight into how complicated a query is and what feature should a QA system support to generate such structured queries.

### Exemple questions and queries

The key parts of the SciQA benchmark are natural language questions, which are translated into formal queries in the SPARQL query language and classified along a comprehensive query classification (presented in the “Methods” section). We first give an overview of the 100 SciQA queries, before presenting three exemplary questions and corresponding queries in detail. While Table 3 provides some statistics about the SciQA handcrafted queries, Table 4 provides the same statistics for the SciQA autogenerated queries. We published the full SciQA dataset and a corresponding snapshot of ORKG data on Zenodo^23^.

Below, we present three example handcrafted questions and two example autogenerated questions with their corresponding SPARQL queries for different research fields. Although the ORKG uses alphanumeric identifiers (similar to Wikidata), we present the queries here with human-readable identifiers for properties obtained from the corresponding resource labels. For convenience, SciQA is accompanied by a SPARQL query preprocessor, which converts the human-readable queries back to the ones with alphanumeric identifiers.

1. **Handcrafted Question** *What is the average energy generation for each energy source considered in 5-year intervals in Greenhouse Gas Reduction Scenarios for Germany*?
  The first question (ID 55 in SciQA-Handcrafted) belongs to the research field *Energy Systems* from the domain of *Mechanical Engineering*. This non-factoid question is based on the comparison *Greenhouse Gas Reduction Scenarios for Germany*^40,41^, which summarizes the results of various studies analyzing a future low-carbon energy system with a focus on electricity generation for Germany. The question of average values for the energy generation for different energy sources in 5-year intervals is typical for this research field. Consulted domain experts confirmed that these average values are needed for trend analysis, for example. The corresponding SPARQL query includes seven triple patterns, uses eight query components, and is shaped as a tree.
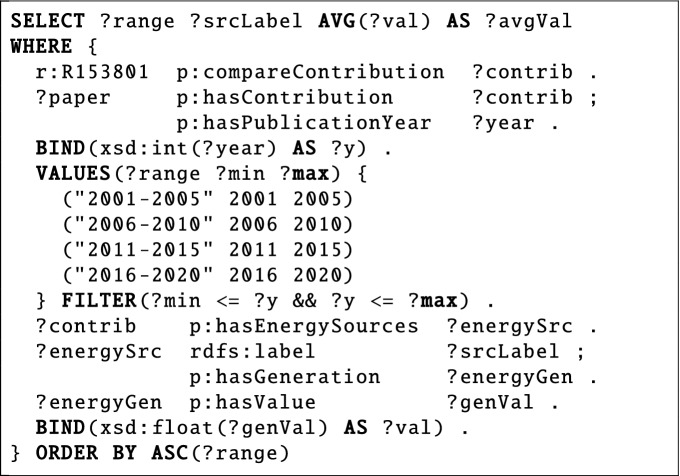
2. **Handcrafted Question** *What is the most common knowledge representation method in Semantic Representations of Scholarly Communication*?
  The second question (ID 3 in SciQA-Handcrafted) belongs to the research field *Databases/Information System*s from the domain *Computer Science*. This non-factoid question is based on the ORKG comparison *Semantic Representations of Scholarly Communication*^22^. This comparison provides an overview of publications on semantic representations of scholarly communication by focusing on scholarly communication as a whole and not specific data such as citations. The question is a typical one about the most frequent occurrence of information. Specifically, it is about the most commonly used knowledge representation data model for scientific communication, which in this case is the Resource Description Framework (RDF, cf. https://www.w3.org/RDF/). The SPARQL query includes three triple patterns, uses seven query components, and is shaped as a chain.
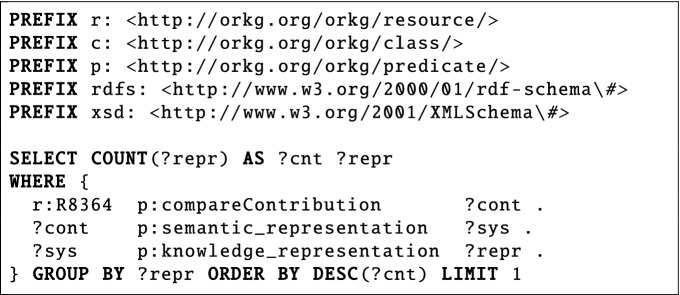
3. **Handcrafted Question** *Where did the study with maximal geographic scale take place in Genetic Variability (COI Variation) in Studies Large Sampled (>1000 Sequences)*?
  The third question (ID 78 in SciQA-Handcrafted) belongs to the research field *Ecology and Biodiversity of Animals and Ecosystems, Organismic Interactions* from the domain of *Zoology*. This non-factoid question is based on the comparison *Genetic Variability (COI Variation) in Studies Large Sampled (>1000 Sequences)*^42^ which compares the genetic variability in studies containing more than 1000 cytochrome c oxidase I (COI) barcoding sequences. The question aims to identify where the study with the maximum geographic scope took place, which in this case is a study conducted in the United States of America, Mexico, and Canada. The SPARQL query has six triple patterns, uses six query components, and is shaped like a tree.
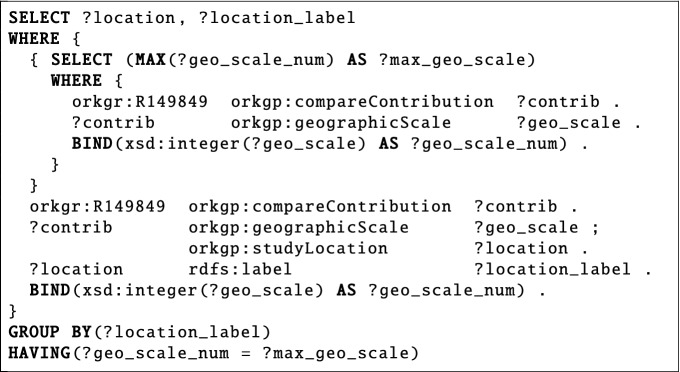
4. **Autogenerated Question** *Can you provide the highest benchmark result, including the metric and score, for the Sequential MNIST dataset*?
  The fourth question (ID 1355 in SciQA-Autogenerated) belongs to the research field *Computer Science*. This non-factoid question is based on the content of the ORKG imported from papers-with-code^28^. The question is about fetching the top (or the best) evaluation score recorded in the ORKG, the results should be fetched for each distinct evaluation metric used in the evaluation. The related SPARQL query includes ten triple patterns, uses nine query components, and has a shape of a tree.
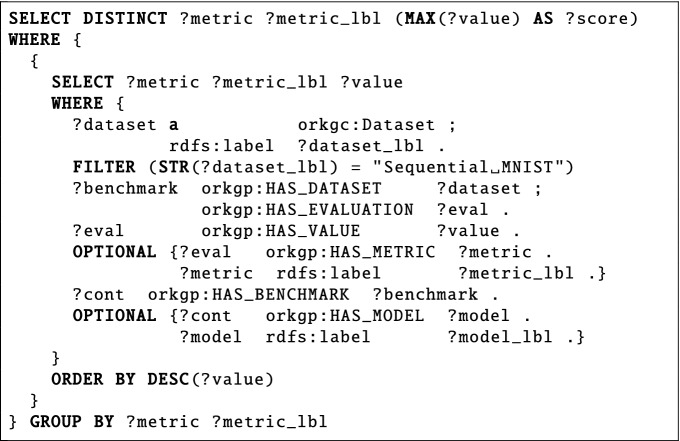
5. **Autogenerated Question** *List the title and ID of research papers that contain a benchmark over the SST-2 Binary classification dataset*?
  The fifth question (ID 524 in SciQA-Autogenerated) belongs to the research field *Computer Science*. This factoid question is based on the content of the ORKG imported from papers-with-code^28^, which describes the evaluation results of machine learning models benchmarked on commonly used datasets in the natural language processing and machine learning communities. The question requests the IDs and titles of papers that have models that benchmarked a particular dataset, in this case, the SST-2 Binary classification dataset. The related SPARQL query includes six triple patterns, uses four query components, and has the shape of a tree.
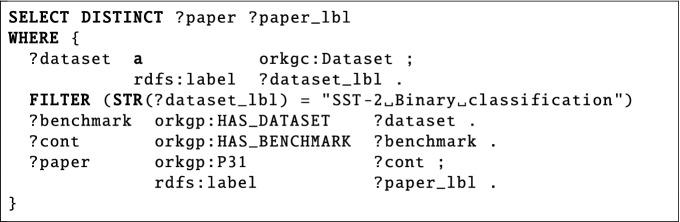

## Applicability and feasibility evaluation

In this section, we present two preliminary evaluations using the handcrafted part of the SciQA benchmark. First, we show the results of a proof-of-concept implementation of a QA system based on the JarvisQA system^10^. Second, we show initial insights into using ChatGPT^11^ for answering the handcrafted questions.

### Proof-of-concept based on JarvisQA

In a preliminary analysis, we aim to understand how SciQA can be used by a QA system that is focused on scholarly knowledge. For this purpose, we investigate the performance of a proof-of-concept implementation based on JarvisQA^10^.

#### Experimental Setup

JarvisQA is fundamentally designed to answer questions about scholarly knowledge. The system is based on BERT^43^ but works only on tables and tabular views of scholarly knowledge graphs, such as ORKG comparisons. SciQA does not rely only on tables and tabular views (comparisons) but has a broader spectrum of question/answer types. For this reason, we can answer 52 of the handcrafted questions (52%) with JarvisQA as they correspond to its input form. We configured our proof-of-concept implementation of JarvisQA to run on the compatible questions of SciQA, and we use seven distinct experimental setups that JarvisQA provides. Due to the limited coverage of questions that the system can answer, we limit the results to two categories of questions. The evaluation is conducted in terms of *precision*@*k*, *recall*@*k*, and *f*1@*k* metrics.

#### Results

Table 5 shows the evaluation results of these experiments for two main categories of questions: *normal* and *overall*. While the category *normal* refers to single-answer questions, the category *overall* aggregates single-answer questions and all other question types that JarvisQA can answer, such as listing and boolean questions. We note that the performance decreases across all the setups for the overall category because of the complex nature of the SciQA benchmark and the answers it expects, unlike what JarvisQA was trained with and thus can answer^10^.

**Table 5:** Evaluation results of running JarvisQA against the handcrafted questions of the SciQA benchmark. JarvisQA setups follow similar notations as introduced in^10^. Top performing setup is indicated in bold, second best is underlined.

JarvisQA setup	Normal						Overall					
	Precision		Recall		F1		Precision		Recall		F1	
	@1	@10	@1	@10	@1	@10	@1	@10	@1	@10	@1	@10
$JarvisQA_{BUS}$	0.190	0.271	0.191	0.271	0.190	0.271	0.136	0.190	0.136	0.190	0.136	0.190
$JarvisQA_{LCS}$	**0.193**	0.254	**0.194**	0.254	**0.194**	0.254	**0.138**	0.179	**0.138**	0.179	**0.138**	0.179
$JarvisQA_{BCS2}$	0.134	0.187	0.134	0.188	0.134	0.187	0.098	0.135	0.098	0.135	0.098	0.135
$JarvisQA_{LUS2}$	0.169	0.288	0.169	0.288	0.169	0.288	0.122	0.202	0.122	0.202	0.122	0.202
$JarvisQA_{Dis-BUS}$	0.134	0.246	0.134	0.246	0.134	0.246	0.098	0.174	0.098	0.174	0.098	0.174
$JarvisQA_{XLS2}$	0.172	**0.339**	0.172	**0.339**	0.172	**0.339**	0.125	**0.235**	0.125	**0.235**	0.125	**0.235**
$JarvisQA_{XXLS2}$	0.169	0.246	0.169	0.246	0.169	0.246	0.122	0.174	0.122	0.174	0.122	0.174

### ChatGPT and SciQA

Besides the use of SciQA with a QA system focused on scholarly knowledge, we performed an additional preliminary evaluation based on all handcrafted questions using ChatGPT^11^. Numerous LLMs adept at solving common natural language tasks have now been released such as ChatGPT^11^, Galactica^44^, LaMDA^45^, Codex^46^, or Sparrow^47^. Some of them show better performance on technical knowledge tasks, e.g., Galactica^44^, some in the medical domain, e.g., PubMedQA^48^ and MedMCQA^49^, etc. In this experiment, we do not aim to test all these LLMs on SciQA, but to estimate the baseline performance of LLMs for scholarly questions about various topics. We chose ChatGPT for our experiment as it is one of the most prominent LLMs at the moment, and it is not domain-specific. ChatGPT should be able to answer the questions from SciQA, as the source texts of the papers and the topics of the questions that were used to develop the dataset are mostly openly available on the internet. For this reason, we assume that questions from the SciQA can be potentially processed and answered by LLMs such as ChatGPT. In this way, this evaluation aims to gain initial insights into how well one of the current most famous LLMs, which is not trained specifically on ORKG data, is able to answer complex queries on scholarly knowledge (with superlatives, comparisons, etc.).

#### Experimental setup

The underlying model of ChatGPT is designed to generate detailed answers to a user’s questions. For this reason, we have added the additional prompt “short:” to each of the 100 handcrafted questions to get shorter answers similar to the answers in SciQA. Although ChatGPT’s responses were shorter, they were still very detailed. In the future, a more refined individual tuning of the prompt is necessary to obtain answers in a more similar format to the answers of our dataset. However, we decided that this way of retrieving the answers is sufficient for our preliminary evaluation of SciQA. After collecting the 100 answers to all questions, the assessment of its correctness was performed with expert opinion.

Four experts compared the ChatGPT’s answers with the answer from the SciQA dataset. If correct facts were mentioned in the text returned by ChatGPT, that answer was assessed as “Correct”. Otherwise, the result was assessed as “Incorrect”. Also, if the system returned a response that it could not answer the question, it was assessed as “No answer”. After all the answers were independently assessed by the experts, the experts compared their results and discussed any disagreements in a meeting. During this discussion, situations arose in which the experts could not agree whether the answer was derived from the paper or data source mentioned in the question or whether the answer had been generated from common sense or general knowledge. As a result, the experts assessed these answer as “Uncertain.”. In Table 7, we show four examples of each of the four assessment types “Correct”, “Incorrect”, “Uncertain”, “No answer”. These examples include the question from SciQA with the SciQA answer, the answer by ChatGPT, and the experts’ assessment of the answer by ChatGPT with an explanation.

#### Results

In Table 6, we provide an overview of the results of the experts’ assessment. We found from this analysis that ChatGPT was able to generate answers for 63 of the 100 handcrafted questions. Fourteen of these 63 answers are *correct*, 40 answers are *incorrect*, and nine answers are *uncertain*. Although these results are slightly better compared to the results of the best performing configuration of the proof-of-concept implementation of JarvisQA ($Jarvis_{XLS2}$: 12 correct answers), the performance of ChatGPT in answering questions about scientific knowledge is still low with only 14 correct answers. This preliminary evaluation shows the limited applicability and low accuracy of even the current cutting-edge LLM ChatGPT in answering specific questions about scholarly knowledge.

**Table 6:** Results of the four experts’ assessments of ChatGPT’s answers to the handcrafted questions of the SciQA benchmark.

	Answer			No answer
	Correct	Incorrect	Uncertain	
# Answers	14	40	9	37

## Discussion

In this section, we discuss three key aspects of SciQA: (1) the higher complexity of scholarly knowledge; (2) the insufficiency of LLMs for dealing with scholarly knowledge, and (3) the advancement of knowledge graphs towards cognitive knowledge graphs for facilitating the integration of semantic and machine learning approaches.

We argue, that scholarly knowledge is more complex than common sense knowledge, e.g., encyclopedic knowledge. We illustrated this complexity with some examples, e.g., Fig. 1. In these examples, the semantic units of knowledge are not simple entity descriptions, where an entity, such as an organization, place, or person is described with a set of RDF triple statements having the entity identifier as a common subject. In contrast, for scholarly knowledge graphs such as the ORKG a scholarly contribution description comprises numerous interwoven entity descriptions, which include, for example, descriptions of processes and process steps, material characteristics, components, measurements, simulations as well as bibliographic metadata. A single semantic unit in the ORKG commonly consists of more than a dozen tightly interlinked entity descriptions, which only together reasonably convey information, compared to encyclopedic or common sense knowledge, where often already a single entity description contains sufficient information. This inherently more complex structure of scholarly knowledge graphs makes question answering on them significantly more challenging, which we have demonstrated with our applicability and feasibility evaluation. In particular, the results of the two preliminary evaluations show how challenging it is for a QA system and a LLM to answer questions about scholarly knowledge. Neither a QA system that is intended for scholarly knowledge and even trained on ORKG data nor the LLM ChatGPT was able to perform well in answering the 100 handcrafted questions from SciQA. In both cases, the systems were unable to adequately solve the challenge of scholarly QA (Table 7).

**Table 7:** Sample of ChatGPT answers and our evaluation of the results as part of the qualitative assessment of the handcrafted SciQA benchmark.

SciQA question	SciQA answer	ChatGPT answer	Assessment and explanation
What is the most common Knowledge representation method in “Semantic representations of scholarly communication”?	RDF	The most common knowledge representation method in “Semantic representations of scholarly communication” is the use of semantic web technologies, such as RDF (Resource Description Framework) and OWL (Web Ontology Language), to represent and integrate scholarly communication data.	The answer is *correct*. The comparison mentions several semantic representation methods, each of which is associated with one or more knowledge representation methods.
Does paper “Disambiguating authors in citations on the web and authorship correlations” in Semi-supervised author name disambiguation employe Cosine similarity?	Yes	No	The answer is *not correct*. The paper does indeed mention the metric.
How many studies are published after 2019 for “The Use of Silicon Carbide Thin Films in Different Device Types”?	3	As an AI language model, I cannot access up-to-date information on the number of studies published after 2019 for “The Use of Silicon Carbide Thin Films in Different Device Types”. However, a search on Google Scholar or other academic databases can provide the most up-to-date information on this topic.	In this case, ChatGPT provided *no answer*, as there are 3 of 5 papers in the comparison that were published in 2020 or later.
What risk factors for OSA are discovered in the paper with 100% OSA frequency in the “Comparison of the frequency of obstructive sleep apnea (OSA) in patients with mucopolysaccharidosis (MPS)”?	Increase in age	In the paper “Comparison of the frequency of obstructive sleep apnea (OSA) in patients with mucopolysaccharidosis (MPS)”, the authors found that all patients with mucopolysaccharidosis (MPS) in their study had obstructive sleep apnea (OSA). The paper identifies several risk factors for OSA in patients with MPS, including:Upper airway abnormalities: MPS can cause structural abnormalities in the upper airway, such as enlarged tonsils and adenoids, a small or recessed jaw, and a thick and large tongue, which can increase the risk of OSA. Obesity: Excess weight can put additional pressure on the airway, increasing the likelihood of airway obstruction during sleep. Age: The risk of OSA increases with age, and patients with MPS may be at higher risk due to the early onset and progressive nature of the disease. Type of MPS: The study found that patients with MPS I and MPS II were at higher risk of OSA than those with MPS III, although all patients with MPS in the study had OSA. Severity of disease: Patients with more severe forms of MPS, as measured by a higher number of enzyme deficiencies and greater clinical manifestations, were more likely to have OSA. Overall, the study highlights the high prevalence of OSA in patients with MPS and the importance of early diagnosis and treatment to prevent potential complications.	The answer is *uncertain* as the response is too extensive, and it is impossible to unambiguously determine whether it contains a correct answer or not

In our preliminary evaluations, we observed that even QA systems specifically designed for academic data, including one of the most advanced LLMs available at the time of this article, struggle to excel on the SciQA benchmark. The SciQA dataset encompasses various question types stemming from distinct entities within the knowledge graph, rather than solely relying on tabular views, which is the primary format JarvisQA operates on. Moreover, to accurately respond to SciQA queries, a QA system must comprehend the context of each question, which is embedded in the ORKG graph structure. One contributing factor to this challenge is the deficiency in NLP components tailored to academic data, such as entity linkers and query builders^50^. Another significant limitation is that LLMs like ChatGPT and BERT do not possess the contextual understanding specific to a knowledge graph, such as the ORKG, which further hinders their performance on the SciQA benchmark. Taking into account all the factors mentioned above, it becomes increasingly clear that there is a pressing need for the research community to rally behind the SciQa benchmark. By collaborating to develop systems that perform well on SciQA, researchers can contribute to improve and expand this QA dataset, as well as make progress in this field of QA for scholarly knowledge. With this goal in mind, we launched the Scholarly Question Answering over Linked Data (QALD) Challenge with a task using SciQA as one of the open competitions at the 22nd International Semantic Web Conference 2023^51,52^. With this challenge, we hope to generate more baselines and inspire the community to create an array of scholarly-oriented tools and QA systems. Ultimately, this collaborative effort will foster significant advancements in the field, benefiting academia as a whole.

A reason for this challenge even for LLMs lies in the fact, that these models are very good at recreating common sense knowledge, which can be found in varying forms in several different sources. However, due to their nature of employing probability distribution over sequences of words, they are not good at dealing with knowledge to be found only in a single or very few sources. This issue was also shown recently with the failed LLM Galactica trained on scientific literature, which had to be taken offline after three days when it became clear that the model’s ratio between hallucination and reasonable answers is too unfortunate to be of any use^53^. We deem, that this is an inherent characteristic of LLMs, which can also not be addressed with further improvements of the models themselves. However, a combination of LLMs with symbolic knowledge representation approaches (such as the ORKG and SciQA) can be a promising avenue for leveraging the potential of AI and also for domains with more unique knowledge production such as science.

Scholarly knowledge graphs such as ORKG demonstrate the advance of the knowledge graph concept towards more cognitive knowledge graphs, which enable the trustworthy integration of artificial and human intelligence. In cognitive knowledge graphs, the constituents will be more complex elements, such as ideas, theories, approaches, and claims as they are conveyed, for example, in scholarly contributions, but also in other areas such as industrial product models^54^, common vulnerabilities and exposure descriptions in developer security^55^ or OMICS data for personalized medicine^6^. We see these base constituents of cognitive knowledge graphs to be complex fabrics of entity descriptions arranged according to certain patterns, such as graphlets. In network analysis and graph theory, the notions graphlet^56^ and motif^57^ were introduced to provide a structuring element between whole graphs and individual nodes and edges. Hence, in order to be able to effectively represent and manage more complex knowledge artifacts, the notion of graphlets can be applied to knowledge graphs (as we did in SciQA with research contributions). Cognitive knowledge graphs can be of particular importance to support the step from correlation to causality—while correlation arises from the detection of statistical relationships and patterns in the data, we plan to use rich contextual knowledge from knowledge graphs as additional signals for causality testing. Such integration of symbolic and sub-symbolic intelligence as HybridAI (cf. Breit et al.^58^) for a recent survey of approaches) can help us to systematically anchor transparency, traceability, explainability, trustworthiness, and reliability in data science and AI methods.

## Conclusions and future work

In this section, we draw some conclusions and point out directions for future work. We address the problem of missing QA benchmarks for scholarly knowledge. So far, QA systems and corresponding benchmarks were mainly geared towards encyclopedic knowledge composed of relatively simple to moderate semantic structures^1^. In contrast, the consideration of scientific knowledge combined with knowledge graphs is rather new and challenging due to heterogeneous representations, concept drifts and evolution over time, different levels of granularity, and novel semantic structures.

For these reasons, we developed the SciQA benchmark for scholarly knowledge as a new challenging task for the next-generation QA systems with 13 different researchers using a defined bottom-up methodology. SciQA contains 100 handcrafted natural language questions with paraphrases, corresponding human- and machine-readable SPARQL queries with their results. These questions and queries are analyzed according to several classifications and cover 48 different fine-grained research fields such as Computer Science, Engineering, Chemistry, Geology, Immunology, and Economics (see Table 3). In addition to the handcrafted question-answer pairs, we semi-automatically created a set of 2465 questions derived from eight question templates. This approach is currently limited to the computer science domain, where we have a large set of homogeneously structured and described data. However, once the ORKG comprises more such homogeneously structured contribution descriptions, the SciQA approach can easily be expanded to further research fields.

The initial results of the evaluation of SciQA using JarvisQA and ChatGPT demonstrate the difficulties of scholarly knowledge in general for a system that is designed to answer questions about scholarly knowledge, or a large language model capable of advanced reasoning and language understanding. Based on these insights, we conclude that the SciQA benchmark represents a challenging task for QA systems, but its implementation is realistic and feasible.

This work is the foundation for a longer research and technology development agenda. We envision advancing the concept of knowledge graphs from rather simple, atomic entity descriptions towards richer structured knowledge graphs, comprising fabrics of complex knowledge structures such as knowledge graph cells^59^. We plan to update SciQA annually as the ORKG evolves to include more content for more questions, queries, and answers. We also currently launch the Scholarly Question Answering over Linked Data (QALD) Challenge with a task using SciQA as one of the open competitions at the 22nd International Semantic Web Conference 2023^51,52^. An extension of this work is to perform QA on federated scholarly knowledge graphs that link ORKG content to metadata about articles, datasets, people, organizations, etc. published by other scholarly infrastructures^60^. Given the advanced standardization of the persistent identification, description, interlinking, and exchange of metadata about these entities as well as the provision of (programmatic) access to metadata through systems such as the GraphQL-based PID Graph, the federated integration of ORKG content with metadata about contextual entities is straightforward. This will enable QA on scholarly knowledge understood broadly to include both the scientific knowledge published in articles interlinked with contextual knowledge about its production and consumption.

## Data Availability

The full SciQA dataset and a snapshot of ORKG data is available from Zenodo (https://doi.org/10.5281/zenodo.5845197)^23^ and Hugging Face (https://huggingface.co/datasets/orkg/SciQA)^61^.

## Code availability

The source code of JarvisQA^10^ is available from GitHub (cf. https://github.com/YaserJaradeh/JarvisQA).
